# Human Amniotic Epithelial Cells as a Tool to Investigate the Effects of Cyanidin 3-*O*-Glucoside on Cell Differentiation

**DOI:** 10.3390/ijms22073768

**Published:** 2021-04-05

**Authors:** Shinya Takahashi, Farhana Ferdousi, Yun-Wen Zheng, Tatsuya Oda, Hiroko Isoda

**Affiliations:** 1 Faculty of Life and Environmental Sciences, University of Tsukuba, Tsukuba 305-8572, Japan; takahashi.shinya.fp@u.tsukuba.ac.jp (S.T.); ferdousi.farhana.fn@u.tsukuba.ac.jp (F.F.); 2 Alliance for Research on the Mediterranean and North Africa (ARENA), University of Tsukuba, Tsukuba 305-8572, Japan; 3 Open Innovation Laboratory for Food and Medicinal Resource Engineering (FoodMed-OIL), AIST-University of Tsukuba, Tsukuba 305-8565, Japan; ywzheng@md.tsukuba.ac.jp (Y.-W.Z.); tatoda@md.tsukuba.ac.jp (T.O.); 4 Department of Gastrointestinal and Hepato-Biliary-Pancreatic Surgery, Faculty of Medicine, University of Tsukuba, Tsukuba 305-8575, Japan

**Keywords:** amniotic epithelial cells, cyanidin-3-*O*-glucoside, global gene expression, cell differentiation, adipocyte

## Abstract

Cyanidin, a kind of anthocyanin, has been reported to have chemotherapeutic activities in humans. Human amniotic epithelial cells (hAECs) are considered a potential source of pluripotent stem cells. hAECs have been used as a novel tool in regenerative cellular therapy and cell differentiation studies. In this study, to explore the effects of cyanidin-3-*O*-glucoside (Cy3G) on hAECs and their mechanisms, we investigated the transcriptomic changes in the Cy3G-treated cells using microarray analysis. Among the differentially expressed genes (Fold change > 1.1; *p*-value < 0.05), 109 genes were upregulated and 232 were downregulated. Ratios of upregulated and downregulated genes were 0.22% and 0.47% of the total expressed genes, respectively. Next, we explored the enriched gene ontology, i.e., the biological process, molecular function, and cellular component of the 37 upregulated (>1.3-fold change) and 124 downregulated (<1.3-fold change) genes. Significantly enriched biological processes by the upregulated genes included “response to muscle activity,” and the genes involved in this gene ontology (GO) were *Metrnl* and *SRD5A1*, which function in the adipocyte. On the other hand, the cell cycle biological process was significantly enriched by the downregulated genes, including some from the *SMC* gene family. An adipogenesis-associated gene *DDX6* was also included in the cell cycle biological process. Thus, our findings suggest the prospects of Cy3G in modulating adipocyte differentiation in hAECs.

## 1. Introduction

In recent years, demands in foods rich in phytochemicals have increased [1]. Based on considerable epidemiological evidence, the presence of polyphenols has been proven to promote health and diminish the risk of various diseases such as cancers and cardiovascular diseases, etc. [2,3]. Polyphenols are contained in various vegetables and fruits [4]. Diets rich in polyphenols lead to the reduction of risks of several diseases [5,6]. To investigate the unknown functions of several phytochemicals beneficial to human health, employment of high-throughput systems based on mammalian cells is one of the best ways.

Human amniotic epithelial cells (hAECs) are considered one of the novel sources of pluripotent stem cells (PSCs). In recent years, growing attention has been given to the use of perinatal stem cells such as hAECs in the field of regenerative cellular therapy and cell differentiation studies. hAECs have some advantages over other PSCs in that they are isolated from discarded term placenta, which is a medical waste product and have similar pluripotent and multipoint properties of stem cells [7]. Therefore, hAECs are suitable stem cell sources with therapeutic applications. Although the effects of plant-derived compounds are extensively studied in PSCs and adult stem cells, studies on hAECs using natural compounds are very scarce. Recently, we have reported that verbenalin, a kind of phytochemical, has exerted therapeutic potential for Alzheimer’s disease in hAECs [8]. Microarray gene expression profiling showed that rosmarinic acid- and isorhamnetin-treated hAECs exerted neuronal and hepatic-lineage specific differentiation potential respectively [9,10]. A caffeoylquinic acid derivative, namely 3,4,5-Tri-*O*-Caffeoylquinic Acid, has also enhanced neural and pigment cell differentiation in hAECs [11]. Therefore, hAECs can be used to explore novel functions of natural compounds. Moreover, priming of hAECs with natural compounds may enhance their differentiation direction towards expected cell lineage; in addition, pretreatment with polyphenols may improve the cell’s functional activities. In this regard, we have investigated the effect of cyanidin on gene expression profiling of hAECs.

Anthocyanins are water-soluble and glycosylated forms of blue, red, or purple-colored pigments found in fruits and vegetables, such as berries, grapes, some tropical fruits as well as red to purplish blue-colored leafy vegetables, colored wheat grains, and roots [12]. Anthocyanins possess anti-inflammatory, antioxidant, antidiabetic, anticancer, antiobesity, and cardioprotective effects [13]. Cyanidin, one of the anthocyanins, has a flavone skeleton with glucoside and is widely distributed in several vegetables and fruits, red leaf lettuce, black soybean, blackberries, and grapes [14,15]. Several kinds of cyanidin have been reported to have chemotherapeutic activities in humans. Cyanidin-3-*O*-β-glucopyranoside is known to induce apoptosis in T-lymphoblastoid and differentiation in HL-60 promyelocytic cells [16,17]. Cyanidin-3-*O*-glucoside (Cy3G) is the major anthocyanin found in most plants and confers a red hue to fruits [18]. Cy3G exerts high antioxidant activity [18]. It is also reported to enhance exercise performance by developing beige fat cells in mice [19,20]. In cell-based research, Cy3G treatment inhibited interleukin-6 production, an inflammatory mediator, in adipose stem cells [21]. There are a few reports on the effects of Cy3G on differentiation promotion of stem cells. In osteoclasts, cyanidin has dual effects on RANKL-induced osteoclastogenesis, differentiation, and fusion [22]. One study has reported chondrogenesis-stimulating effects of cyanidin on adipose tissue derived-human mesenchymal stem cells (MSCs) [23]. Cyanidin treatment also inhibited chondrogenic and hypertrophic differentiation of MSCs via decreasing autophagic activity [24]. However, studies on the effects of cyanidin on stem cell differentiation are still limited, and there is no report on this compound’s effect on hAECs. In this study, we investigated the transcriptomic changes in the Cy3G-treated hAECs to explore its effects and probable mechanism on hAECs. From our results, we can anticipate the possibilities of adipocyte differentiation-inducing effects of Cy3G in hAECs.

## 2. Results

### 2.1. Characteristics of Gene Expression Profiling in hAECs

**Figure 1:**
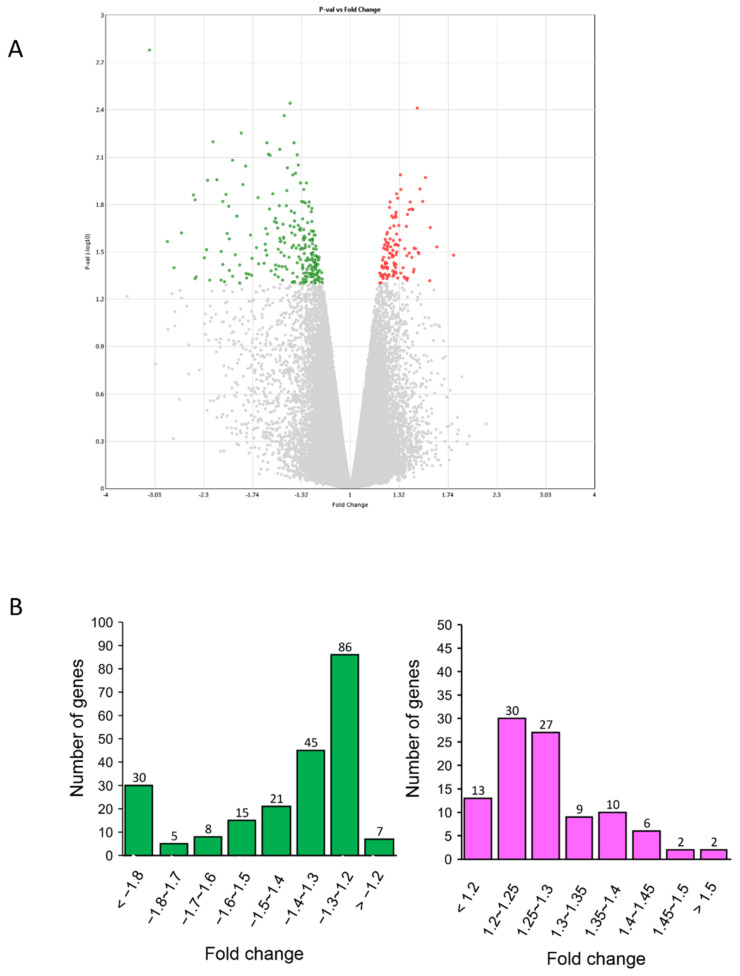
Characterization of gene expression profiles in human amniotic epithelial cells (hAECs) with or without cyanidin-3-*O*-glucoside (Cy3G) (**A**) Volcano plot displaying differentially expressed genes (DEGs) between Cy3G-treated and untreated hAECs on day 7. The vertical axis corresponds to −log10 *p*-value of the ANOVA *p*-values, and the horizontal axis displays linear fold change. The red dots represent the upregulated genes, and the green dots represent the downregulated genes. (**B**) distribution of fold changes of downregulated genes (left) and upregulated genes (right) in Cy3G-treated hAECs.

After seven days of Cy3G treatment, a total of 341 genes were differentially expressed, having fold change >1.1 and *p*-value <0.05 compared to untreated control. Among them, 109 genes were upregulated and 232 were downregulated. Ratios of upregulated and downregulated differentially expressed genes (DEGs) were 0.22% and 0.47% of total expressed genes, respectively (Figure 1A).

### 2.2. Changes of Gene Expression in Cy3G-Treated hAECs

Five upregulated genes showed changes in the expression more than 1.45-fold (Table 1). To know the detailed features of their encoded proteins, we checked their function in the UniProt database (https://www.uniprot.org/, accessed on 16 April 2020).

The highest expressed gene was fructosamine 3 kinase-related protein (*FN3KRP*), which is involved in protein deglycation by mediating phosphorylation of fructoselysine residues on glycated protein to generate fructoselysine-3 phosphate [25]. Another important upregulated gene was meteorin-like protein (*METRNL*), which is a hormone and is induced following exercise or cold exposure and promotes energy expenditure. It is also able to stimulate energy expenditure associated with the browning of white fat depots and thus improves glucose tolerance [26]. Ras-related protein Rab-6A (*RAB6A*) is a regulator of membrane traffic from the Golgi apparatus towards the endoplasmic reticulum and has a low GTPase activity. From gene ontology, RAB6A was also predicted to bind to myosin V [27]. *PSMB8* (20S proteasome subunit beta type-8) has an ATP/ubiquitin-dependent proteolytic activity and processes the class I MHC peptides as the immunoproteasome [28]. *DCTN4* (dynactin subunit 4), which is a dynactin subunit p62, has been identified as an interacting partner of the P-type ATPase, ATP7B protein in mammals [29].

On the other hand, 19 downregulated genes showed fold change more than −2.0 (Table 2). Among the downregulated genes, *DDX6* (DEAD box helicase 6), plays a role in the process of mRNA degradation [30]. The encoded protein of *ZC3H11A* (Zinc finger CCCH domain-containing protein 11A) is involved in nuclear mRNA export [31]. *PRDM2* (PR-domain zinc finger protein 2) encoded protein is an *S*-adenosyl -*L*-methionine-dependent histone methyltransferase that explicitly methylates Lys-9 or histone H3 and may function as a DNA-binding transcription factor [32].

### 2.3. Significantly Enriched Gene Sets and Gene Ontologies (GO)

For the GO analysis, we considered the genes with fold change >1.3 and *p*-value < 0.05 as the differentially expressed genes (DEGs) (Table 1). Upregulated DEGs (>1.3-fold change) were 37 (23%) and downregulated DEGs (1.3-fold>) were 124 (57%) (Figure 1B).

**Figure 2:**
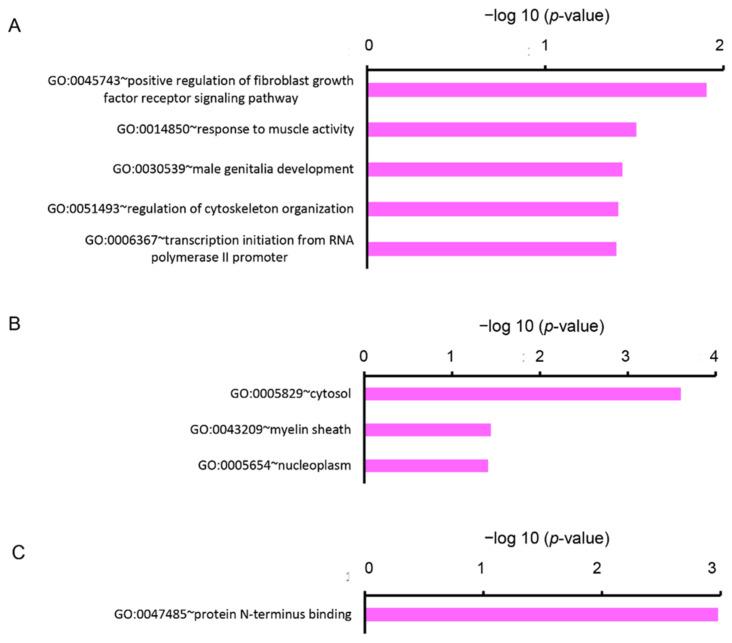
Functional analysis of upregulated DEGs between Cy3G-treated and untreated hAECs on day 7 represented (**A**) significantly enriched biological process, (**B**) cellular components, and (**C**) molecular functions analyzed by the Database for Annotation, Visualization, and Integrated Discovery (DAVID). The horizontal axis corresponds to −log10 *p*-value.

We analyzed the DEGs by enrichment analysis using the Database for Annotation, Visualization, and Integrated Discovery (DAVID) online tool. The upregulated DEGs were categorized into nine clusters based on GO terms of biological process (BP), eight clusters based on cellular components (CC), and three clusters based on molecular functions (MF). In the BP, five gene sets were significantly enriched (*p* < 0.05), containing the GO terms “positive regulation of fibroblast growth factor receptor signaling pathway (GO:0045743)”, “response to muscle activity (GO:0014850)”, “male genitalia development (GO:0030539)”, “regulation of cytoskeleton organization (GO:0051493)”, and “transcription initiation from RNA polymerase II promoter (GO:0006367)” (Figure 2A). In the CC, three gene sets were significantly enriched (*p* < 0.05), containing GO terms “cytosol (GO:0005829)”, “myelin sheath (GO:0043209)”, and “nucleoplasm (GO:0005654)” (Figure 2B). In the MF, one gene set, containing a term “protein N-terminus binding (GO:0047485)” was significantly enriched (*p* < 0.05) (Figure 2C).

**Figure 3:**
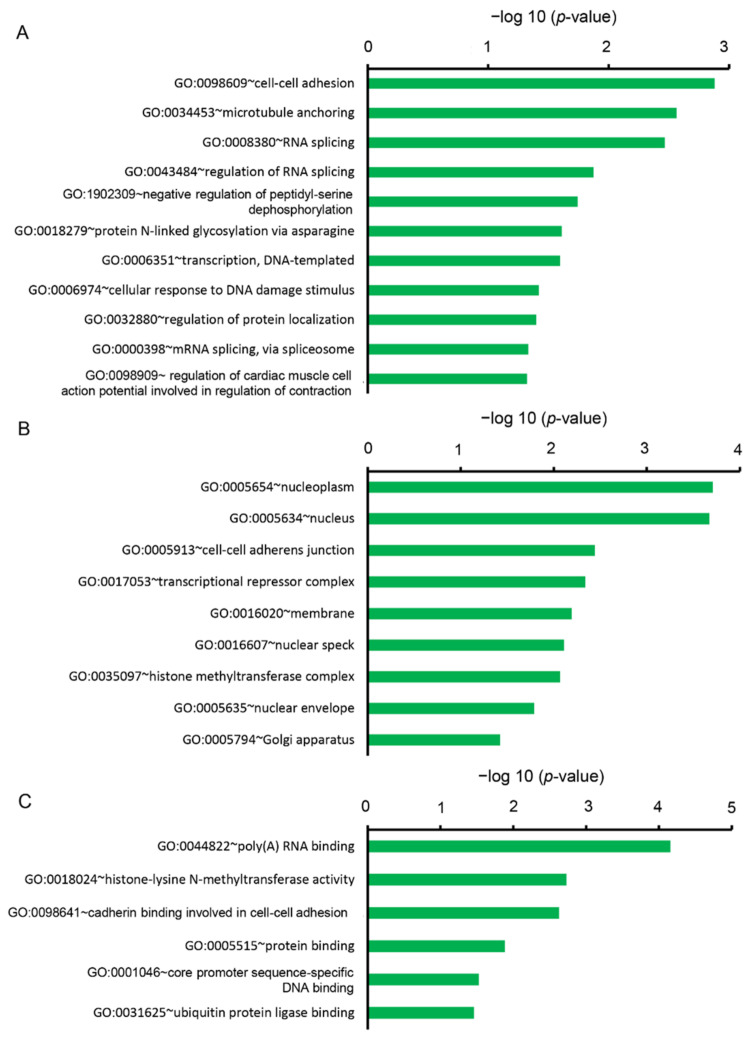
Functional analysis of downregulated DEGs between Cy3G-treated and untreated hAECs on day 7 by DAVID represented (**A**) significantly enriched biological process, (**B**) cellular components, and (**C**) molecular functions. The horizontal axis corresponds to −log10 *p*-value.

On the other hand, the downregulated DEGs were categorized into 22 clusters based on BP, 14 clusters based on CC, and 13 clusters based on MF. In the BP, 11 gene sets were significantly enriched (*p* < 0.05), containing GO terms “cell-cell adhesion (GO:0098609)”, “microtubule anchoring (GO:0034453)”, “RNA splicing (GO:0008380)”, “negative regulation of peptidyl-serine dephosphorylation (GO:1902309)”, “protein *N*-linked glycosylation via asparagine (GO:0018279)”, “transcription, DNA templated (GO:0006351)”, “cellular response to DNA damage stimulus (GO:0006974)”, “regulation of cardiac muscle cell action potential involved in the regulation of contraction (GO:0098909)” (Figure 3A). In the CC, nine gene sets were significantly enriched (*p* < 0.05), containing GO terms “nucleoplasm (GO:0005654)”, “nucleus (0005634)”, “cell-cell adherens junction (GO:0005913)”, “transcriptional repressor complex (GO:0017053)”, “membrane (GO:0016020)”, “nuclear speck (GO:0016607)”, “histone methyltransferase complex (GO:0035097)”, “nuclear envelope (GO:0005635)”, and “Golgi apparatus (GO:0005794)” (Figure 3B). In the MF, six gene sets were significantly enriched (*p* < 0.05), containing GO terms “poly (A) RNA binding (GO:0044822)”, “histone-lysine *N*-methyltransferase activity (GO:0005789)”, “cadherin binding involved in cell-cell adhesion (GO:0098641)”, “protein binding (GO:0005515)”, “core promoter sequence-specific DNA binding (GO:0001046)”, “ubiquitin-protein ligase binding (GO:0031625)”, “chromatin binding (GO:0003682)”, “ATP binding (GO:0005524)”, and “polynucleotide adenyl transferase activity (GO:0004652)” (Figure 3C).

**Figure 4:**
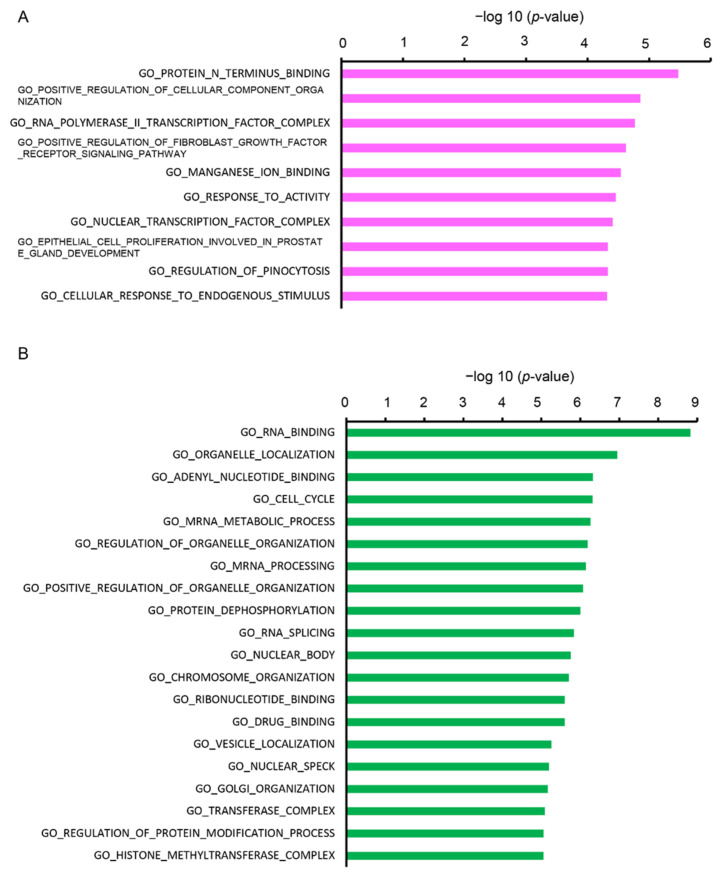
Functional analysis of DEGs between Cy3G-treated and untreated hAECs on day 7 in (**A**) upregulated and (**B**) downregulated genes analyzed by the Molecular Signatures Database (MSigDB). The horizontal axis corresponds to −log10 *p*-value.

Furthermore, we analyzed the DEGs using the Molecular Signatures Database (MSigDB) online tool. The upregulated DEGs were categorized into 10 gene sets and the downregulated DEGs were categorized into 20 gene sets. The top enriched GO terms by upregulated DEGs included “protein N-terminus binding”, “positive regulation of cellular component organization”, “RNA polymerase II transcription factor complex”, and “positive regulation of fibroblast growth factor receptor signaling pathway” (Figure 4A). Top enriched GO by the downregulated DEGs were “RNA binding”, “organelle localization”, “adenyl nucleotide binding”, “cell cycle”, and “mRNA metabolic process” (Figure 4B).

## 3. Discussion

Cyanidin has several bioactivities in mammals. Previous reports indicate that cyanidin may affect the differentiation of PSCs and adult stem cells. Cy3G has been reported to regulate the differentiation of RANKL-induced osteoclasts, which are multi-nucleated cells derived from hematopoietic stem cells (HSCs), and enhance cell fusion [33]. LnCap and DU145 human prostate cancer cells treated with Cyanidin-3-*O*-β-glucopyranoside showed anti-proliferative and pro-differentiation properties [17]. However, the effects of cyanidin on hAECs have not been investigated.

Enrichment analysis of Cy3G-treated hAECs showed that response to muscle activity-related genes, namely *METRNL* and *SRD5A1*, were upregulated (Table 3).

*Metrnl* is reported to have multifaceted functions in adipocyte differentiation concerned to brown fat cell differentiation of adipocyte [34]. Brown adipose tissue can dissipate energy compared to white adipose tissue. White adipose tissue possesses the capacity to generate brown-like adipocytes, termed beiging [35]. *Metrnl* was identified as a peroxisome proliferator-activated receptor gamma coactivator (PGC)-1α4-dependent myokine and can be induced in muscle after exercise. Increased circulating levels of *Metrnl* indicates stimulated energy expenditure and improved glucose tolerance [34]. These changes are activated by the adaptive responses of cells to the environmental or intracellular stress stimuli, thus counteracting the stress and promoting cell survival [36]. Overexpressed *Metrnl* also upregulated the peroxisome proliferator-activated receptor gamma (PPARγ) and anti-inflammatory cytokines in white adipocytes [34].

Interestingly, in the skeletal muscle of Cy3G-administered mice, PGC-1α was upregulated via cyclic AMP (cAMP) elevation, which subsequently enhanced exercise performance [19]. Cy3G also induces the differentiation of the beige phenotypes via elevations of cAMP level, PGC-1α, and PPARγ in mice adipocyte cells [19]. These previous reports support our findings in transcriptome data using Cy3G-treated hAECs.

Another gene, *SRD5A1*, converts testosterone into 5-alpha-dihydrotestosterone and progesterone or corticosterone into their corresponding 5-alpha-3-oxosteroids, and has a central role in sexual differentiation and androgen physiology. Male mouse that lacked 5-alpha-reductase type 1 (SRD5A1) has reduced bone and forelimb muscle grip strength [37]. Expression of *SRD5A1* was found in human adipose tissues, where it shows a tendency to increase slightly with differentiation in preadipocytes [38].

PSMB8, a catalytic subunit for immunoproteasomes, also regulates the differentiation of preadipocytes and the differentiation of preadipocytes to mature adipocytes in mice. *Psmb8−/−* mice show reduced weight gain caused by a reduction in adipose tissue volume and small size of mature adipocytes. In addition, inhibition of Psmb8 in 3T3L1 mice adipocyte cells could disrupt the differentiation to mature cells [39].

In our study, enrichment analysis also showed upregulation of genes related to regulation of cytoskeleton organization such as *CAPN2* and *STMN1* (Table 3). *CAPN2* encodes calpain-2 catalytic subunit, which is a calcium-regulated non-lysosomal thiol-protease. It catalyzes limited proteolysis of substrates involved in cytoskeletal remodeling and signal transduction [40]. *STMN1* encodes Stathmin, which is involved in the regulation of the microtubule filament system by destabilizing microtubules. Cytoskeleton remodeling is one of the first steps for the morphological transition from preadipocyte to mature adipocyte. Acetylation of α-tubulin is related to adipogenesis in mice and 3T3-L1 cells [41].

A downregulated gene, *DDX6*, which encodes DEAD-box helicase 6 protein, has been reported to function in adipogenesis. In human adipose tissue-derived stem cells, the number of DDX6 granules per cell was reduced during adipogenesis [42].

Enrichment analysis showed cell cycle-related genes were downregulated in the Cy3G-treated hAECs. This gene set includes 21 genes as follows: *YMHAE*, *EIF4G1*, *SMC1A*, *TENT4B*, *PRPF40A*, *SON*, *CNOT4*, *WASL*, *AKAP9*, *MEI1*, *GOGGA2*, *CEP57*, *PCM1*, *CDK12*, *SMC5*, *TLK1*, *WAC*, *CBX5*, *RMI1*, *SPIN1*, and *MGA*. *CDK12,* which encodes cycle dependent kinase 12, is associated with elongating RNA polymerase II and its activity is required for G1/S progression [43]. *SMC1A* encodes a protein of central component of chromosome cohesion complex during cell cycles [44]. Cell cycle is an important event during cell proliferation but not necessary during cell differentiation. The length of G1 phase arrest is important for the decision of proliferation or differentiation [45]. In Cy3G- treated AECs, downregulation of genes related to G1/S progression may be indicated of the induction of differentiation in hAECs.

Previously, Kim et al. (2012) reported that anthocyanin extracts from black soybeans comprising Cy3G (68.3%) as well as delphinidin-3-*O*-glucoside (25.2%) and petunidin-3-*O*-glucoside (6.5%) could inhibit adipocyte differentiation and basal lipolysis in 3T3-L1 cell line [46]. Another study by Jeon et al. (2015) reported that the ethanol testa-extract of black soybean might suppress the differentiation of subcutaneous adipose-derived stem cells into the precursor cells of adipocytes [47]. However, adipocyte phenotype was studied in neither of the studies. We have reported in our previous studies that Cy3G, both synthetic and derived from black soybeans, could induce beige phenotype of adipocyte differentiation in mouse preadipocyte 3T3-L1 cells and improves insulin resistance [3,20].

Natural bioactive compounds have great potential to induce the targeted differentiation of stem cells in a lineage-specific manner by modulating cellular behavior and early biological and molecular events; however, only a handful of studies explored it. In our previous studies, we have reported that a caffeic acid ester (rosmarinic acid), a caffeoylquinic acid derivative (TCQA), and an iridoid glycoside (verbenalin) directed the differentiation of hAECs towards neuronal-lineage, whereas a flavonol aglycone isorhamnetin induced hepatic-lineage specific differentiation in hAECs [8,9,10,11]. We assume that other compounds from the anthocyanin group may have a similar effect as Cy3G on hAEC’s fate choice. Their efficacy may vary depending on the presence or absence as well as the position of the sugar moieties. Therefore, the role of different anthocyanins on modulating adipocyte differentiation in hAECs should be explored further. Additionally, assessments of the effects of Cy3G on protein levels is required to confirm our observational transcriptomic analysis findings.

Altogether, our findings suggest that Cy3G may have prospects in inducing adipocyte differentiation in hAECs. Although hAECs have multilineage differentiation potential [7,48], their adipogenic differentiation is still controversial. While some reported adipogenic differentiation of hAECs under proper culture conditions [49,50,51], others did not find any adipogenic differentiation potential of hAECs [52,53]. Therefore, adipocyte differentiation-enhancing effects of Cy3G, a plant-based compound, in hAECs in the absence of any growth factors and cytokines, may have an important step towards clinical applications of hAECs in reconstructive, corrective, and cosmetic fields. However, further in-depth investigation to validate our primary findings is warranted.

## 4. Materials and Methods

### 4.1. Extraction of AECs and Cell Culture Maintenance

Isolation of amnion epithelial cells (AECs) and their culturing was followed by the methods described previously [8,9]. In brief, the amnion was aseptically separated from the chorion and washed with Hank’s Basic Salt Solution -Calcium and Magnesium Free (CMF-HBSS, Wako Pure Chemical Industries Ltd., Osaka, Japan). The smaller pieces of amnion were treated with a pre-digestion buffer (CMF-HBSS with EGTA, Wako Pure Chemical Industries Ltd., Osaka, Japan), rocked in the solution, and incubated for 10 min at 37 °C. After incubation, the Trypsin-EDTA was added to the tissue and incubated for 40 min at 37 °C and then transferred on ice. Dulbecco’s Modified Eagle Medium (DMEM, Sigma-Aldrich, St.-Louis, MI, USA) with FBS (Thermo Fisher Scientific Inc., Waltham, MA, USA) and penicillin-streptomycin (Lonza Walkersville Inc., Walkersville, MD, USA) was added to the trypsin digest. After centrifugation, pellets were resuspended and filtered through a 100 mm filter. The cell suspension was collected.

To maintain the AECs, the cells were cultured in Placenta Epithelial Cell Basal Medium (PromoCell, Cat. #C-26140, Heidelberg, Germany) and monitored continuously with media change every 2–4 days.

### 4.2. Three-Dimension Amnion Epithelial Cells, Culture Spheroid Formation, and Treatment with Cyanidin

To culture the hAECs, we employed the 3D culture Plate system (Elplasia^™^, Kuraray Co. Ltd., Kurashiki, Japan). Spheroids were formed by seeding 1 × 10^6^ AECs in Placenta Basal Epithelial Cell Medium into each well of the 24-well plate. The initial culture was maintained for 24 h. After the initial 24 h culture, the medium was changed with 20 μM of Cy3G (Tokiwa Phytochemical Co. Ltd., Chiba, Japan; purity on HPLC ≥ 98%) every 48 h three times for the treatment samples (Day 2, 4, and 6). Control samples were maintained in the Placenta Epithelial Cell Basal Medium that was also changed every 48 h (Day 2, 4, and 6). Finally, we collected RNA samples from the Cy3G-treated and control AECs on day 7.

### 4.3. RNA Extraction and Microarray Analysis

Total RNA was isolated using ISOGEN (Nippon Gene, Tokyo, Japan), according to the manufacturer’s instructions. The amplified RNA (aRNA) was synthesized using the Gene Chip 3′ IVT PLUS Reagent Kit (Thermo Fisher Scientific Inc., Waltham, MA, USA). Hybridization was achieved using the Affymetrix GeneChip Human Genome U219 Array Strip (HG-U219, Thermo Fisher Scientific). The images were obtained by the GeneAtlas^™^ Imaging Station and analyzed using the GeneAtlas^™^ Workstation (Thermo Fisher Scientific), according to the manufacturer’s instructions. Microarray expression profiling was conducted for two biological replicates of Cy3G-treated samples (Cy7) and untreated control samples (D7).

### 4.4. Microarray Analysis for Gene Expression Profiling in Cy3G-Treated hAECs

The data of DNA microarrays (Cy7 and D7) were classified and analyzed by the gene set enrichment analysis approach, using the DAVID server, v6.8 (National Institute of Allergy and Infectious Diseases (NIAID), NIH, USA, https://david.ncifcrf.gov/, accessed on 16 April 2020) [54,55]. The data sets were also analyzed by the MSigDB v7.1 (Gene Set E nrichment Analysis, UC San Diego, https://www.gsea-msigdb.org/gsea/msigdb/index.jsp, accessed on 16 April 2020).

### 4.5. Ethics Approval

In the present study, we have used hAECs that were preserved at the Tsukuba Human Tissue Biobank Center (THB). THB was established at the University of Tsukuba in November 2013 with an aim to reserve human biospecimens to promote medical research [56,57]. The protocol for isolation, collection, and use of hAECs and other biospecimens was approved by the Ethical Review Committee of the University of Tsukuba Hospital (approval code: H27-58, approval date: 10 July, 2015). Informed written consent was obtained from the mothers who donated the placenta.

## Figures and Tables

**Table 1:** List of upregulated genes with fold change >1.3 compared to control group.

Gene Symbol	Description	Fold Change	*p*-Value
*FN3KRP*	fructosamine 3 kinase-related protein	1.57	0.048
*METRNL*	meteorin-like protein	1.51	0.0152
*RAB6A*	RAB6A, member RAS oncogene family	1.47	0.0319
*PSMB8*	proteasome subunit beta 8	1.46	0.0039
*DCTN4*	dynactin 4 (p62)	1.45	0.0302
*DCAF5*	DDB1 and CUL4 associated factor 5	1.43	0.0171
*NR1H2*	nuclear receptor subfamily 1, group H, member 2	1.43	0.0409
*RAPGEF6*	Rap guanine nucleotide exchange factor 6	1.43	0.0423
*SRD5A1*	steroid-5-alpha-reductase, alpha polypeptide 1 (3-oxo-5 alpha-steroid delta 4-dehydrogenase alpha 1)	1.43	0.0298
*ANKFY1*	ankyrin repeat and FYVE domain containing 1	1.42	0.0343
*NPTN*	neuroplastin	1.4	0.032
*CDIPT*	CDP-diacylglycerol—inositol 3-phosphatidyltransferase	1.39	0.017
*RTCA*	RNA 3′-terminal phosphate cyclase	1.38	0.0477
*STMN1*	stathmin 1	1.38	0.0461
*TAF11*	TAF11 RNA polymerase II, TATA box binding protein (TBP)-associated factor, 28kDa	1.38	0.0335
*WDR1*	WD repeat domain 1	1.38	0.0183
*PPM1B*	protein phosphatase, Mg2+/Mn2+ dependent, 1B	1.37	0.0217
*CAPN2*	calpain 2, (m/II) large subunit	1.36	0.0454
*LEPROTL1*	leptin receptor overlapping transcript-like 1	1.36	0.0302
*UQCRC1*	ubiquinol-cytochrome c reductase core protein I	1.36	0.0463
*GYG1*	glycogenin 1	1.35	0.0397
*GTF2H2*	general transcription factor IIH subunit 2	1.33	0.0127
*MRRF*	mitochondrial ribosome recycling factor	1.33	0.043
*USP6NL*	USP6 N-terminal like	1.33	0.0103
*SIPA1*	signal-induced proliferation-associated 1	1.32	0.0265
*ACLY*	ATP citrate lyase	1.31	0.0145
*DNAJC7*	DnaJ (Hsp40) homolog, subfamily C, member 7	1.31	0.0215
*ID1*	inhibitor of DNA binding 1, dominant negative helix-loop-helix protein	1.31	0.0288
*WDR77*	WD repeat domain 77	1.31	0.0464
*March2*	membrane associated ring finger 2	1.3	0.043
*CTNNB1*	catenin (cadherin-associated protein), beta 1	1.3	0.0136
*GPR150*	G protein-coupled receptor 150	1.3	0.019
*MYH4*	myosin, heavy chain 4, skeletal muscle	1.3	0.0378
*NARF*	nuclear prelamin A recognition factor	1.3	0.0329
*NCOA5*	nuclear receptor coactivator 5	1.3	0.0283
*STRADA*	STE20-related kinase adaptor alpha	1.3	0.0316
*ZNF576*	zinc finger protein 576	1.3	0.0243

**Table 2:** List of downregulated genes with fold change > 1.3 compared to control group.

Gene Symbol	Description	Fold Change	*p*-Value
*DDX6*	DEAD (Asp-Glu-Ala-Asp) box helicase 6	−3.13	0.0017
*ZC3H11A*	zinc finger CCCH-type containing 11A	−2.82	0.0272
*CCDC186*	coiled-coil domain containing 186	−2.73	0.0399
*PRDM2*	PR domain containing 2, with ZNF domain	−2.61	0.0239
*SPAG9*	Sperm-associated antigen 9	−2.43	0.0137
*RBAK*	RB-associated KRAB zinc finger	−2.41	0.0466
*PRRC2C*	proline-rich coiled-coil 2C	−2.4	0.0454
*BOD1L1*	biorientation of chromosomes in cell division 1-like 1	−2.29	0.0345
*BAZ1B*	bromodomain adjacent to zinc finger domain 1B	−2.26	0.0306
*ALG11*	ALG11, alpha-1,2-mannosyltransferase	−2.25	0.0111
*ZNF480*	zinc finger protein 480	−2.22	0.0477
*RNPC3*	RNA binding region (RNP1, RRM) containing 3	−2.18	0.0064
*MIER1*	mesoderm induction early response 1, transcriptional regulator	−2.13	0.011
*PDE4DIP*	phosphodiesterase 4D interacting protein	−2.08	0.0478
*USP1*	ubiquitin specific peptidase 1	−2.08	0.0315
*GCC2*	GRIP and coiled-coil domain containing 2	−2.06	0.0378
*TCERG1*	transcription elongation regulator 1	−2.06	0.0152
*YWHAE*	tyrosine 3-monooxygenase/tryptophan 5-monooxygenase activation protein, epsilon	−2.05	0.0491
*LMAN1*	lectin, mannose-binding, 1	−2.02	0.0137
*CEP57*	centrosomal protein 57 kDa	−2.01	0.0241
*NBPF10*	neuroblastoma breakpoint family, member 10	−1.99	0.0261
*WASL*	Wiskott–Aldrich syndrome-like	−1.99	0.0414
*WHSC1L1*	Wolf–Hirschhorn syndrome candidate 1-like 1	−1.99	0.0163
*SWAP70*	SWAP switching B-cell complex 70 kDa subunit	−1.95	0.0452
*EFCAB14*	EF-hand calcium binding domain 14	−1.9	0.0187
*EIF4G1*	eukaryotic translation initiation factor 4 gamma, 1	−1.88	0.0381
*KMT2C*	lysine (K)-specific methyltransferase 2C	−1.87	0.0496
*TNKS2*	tankyrase, TRF1-interacting ankyrin-related ADP-ribose polymerase 2	−1.86	0.0056
*IL6ST*	interleukin 6 signal transducer	−1.84	0.0118
*SON*	SON DNA binding protein	−1.8	0.0463
*FAM107B*	family with sequence similarity 107, member B	−1.78	0.0437
*SF3B1*	splicing factor 3b, subunit 1, 155 kDa	−1.76	0.0248
*OTUD4*	OTU deubiquitinase 4	−1.75	0.0445
*PPIG*	peptidylprolyl isomerase G (cyclophilin G)	−1.75	0.0348
*PEG10*	paternally expressed 10	−1.71	0.0298
*CBX5*	chromobox homolog 5	−1.69	0.0143
*KMT2A*	lysine (K)-specific methyltransferase 2A	−1.68	0.0378
*ANKRD12*	ankyrin repeat domain 12	−1.63	0.0372
*AP3M2*	adaptor-related protein complex 3, mu 2 subunit	−1.62	0.0284
*REST*	RE1-silencing transcription factor	−1.62	0.0308
*WAC*	WW domain containing adaptor with coiled-coil	−1.62	0.0225
*CTDSPL2*	CTD small phosphatase like 2	−1.61	0.0333
*DNAJB14*	DnaJ (Hsp40) homolog, subfamily B, member 14	−1.6	0.0244
*ZNF208*	zinc finger protein 208	−1.59	0.0076
*CRCP*	CGRP receptor component	−1.58	0.0169
*MLLT4*	myeloid/lymphoid or mixed-lineage leukemia; translocated to, 4	−1.58	0.0077
*PCM1*	pericentriolar material 1	−1.57	0.0419
*MBNL2*	muscleblind-like splicing regulator 2	−1.55	0.0136
*AKAP9*	A kinase (PRKA) anchor protein 9	−1.53	0.0383
*HSP90B1*	heat shock protein 90kDa beta (Grp94), member 1	−1.53	0.044
*MCFD2*	multiple coagulation factor deficiency 2	−1.53	0.0194
*SMC1A*	structural maintenance of chromosomes 1A	−1.52	0.0205
*SMC5*	structural maintenance of chromosomes 5	−1.52	0.0407
*GOLGA2*	golgin A2	−1.51	0.0456
*LRP10*	LDL receptor-related protein 10	−1.51	0.0223
*PTP4A2*	protein tyrosine phosphatase type IVA, member 2	−1.51	0.0303
*SKI*	SKI proto-oncogene	−1.5	0.0161
*SPIN1*	spindlin 1	−1.5	0.0389
*LGALSL*	lectin, galactoside-binding-like	−1.49	0.0259
*MYH11*	myosin, heavy chain 11, smooth muscle	−1.49	0.0071
*TBC1D23*	TBC1 domain family, member 23	−1.49	0.0272
*DYM*	dymeclin	−1.48	0.027
*GAPVD1*	GTPase-activating protein and VPS9 domains 1	−1.48	0.0319
*PGAM5*	PGAM family member 5, serine/threonine protein phosphatase, mitochondrial	−1.47	0.0211
*SUPT20H*	SPT20 homolog, SAGA complex component	−1.47	0.039
*MGA*	MGA, MAX dimerization protein	−1.46	0.0043
*SGMS2*	sphingomyelin synthase 2	−1.46	0.0256
*ZNF91*	zinc finger protein 91	−1.46	0.0258
*PAPOLA*	poly(A) polymerase alpha	−1.45	0.0277
*PHF24*	PHD finger protein 24	−1.45	0.039
*CNOT4*	CCR4-NOT transcription complex subunit 4	−1.44	0.0162
*KTN1*	kinectin 1 (kinesin receptor)	−1.43	0.0406
*MAP4K5*	mitogen-activated protein kinase 5	−1.43	0.0129
*ZNF766*	zinc finger protein 766	−1.43	0.0093
*CDK12*	cyclin-dependent kinase 12	−1.42	0.0343
*ITGAD*	integrin alpha D	−1.41	0.0217
*MGEA5*	meningioma expressed antigen 5 (hyaluronidase)	−1.41	0.0036
*ATF6*	activating transcription factor 6	−1.4	0.0175
*DNASE1*	deoxyribonuclease I	−1.4	0.024
*PRPF40A*	PRP40 pre-mRNA processing factor 40 homolog A	−1.39	0.0491
*RAB11FIP1*	RAB11 family interacting protein 1 (class I)	−1.39	0.0298
*DDR2*	discoidin domain receptor tyrosine kinase 2	−1.38	0.0287
*EPRS*	glutamyl-prolyl-tRNA synthetase	−1.38	0.0102
*HN1L*	hematological and neurological expressed 1-like	−1.38	0.0201
*MEI1*	meiotic double-stranded break formation protein 1	−1.38	0.043
*PAPD5*	PAP-associated domain containing 5	−1.38	0.0489
*PIK3C2A*	phosphatidylinositol-4-phosphate 3-kinase, catalytic subunit type 2 alpha	−1.38	0.0064
*UBE2J1*	ubiquitin-conjugating enzyme E2, J1	−1.38	0.0219
*UBXN4*	UBX domain protein 4	−1.38	0.0492
*CCDC50*	coiled-coil domain containing 50	−1.37	0.0247
*GCK*	glucokinase (hexokinase 4)	−1.37	0.0178
*PLA2G5*	phospholipase A2, group V	−1.37	0.01
*PTPRE*	protein tyrosine phosphatase, receptor type, E	−1.37	0.0491
*ARHGAP35*	Rho GTPase activating protein 35	−1.36	0.0299
*PRKAR2A*	protein kinase, cAMP-dependent, regulatory, type II, alpha	−1.35	0.0455
*TLK1*	tousled-like kinase 1	−1.35	0.0076
*AFF1*	AF4/FMR2 family, member 1	−1.34	0.0214
*LINC00303*	long intergenic non-protein coding RNA 303	−1.34	0.0341
*OR5AK2*	olfactory receptor, family 5, subfamily AK, member 2	−1.34	0.0287
*PCLO*	piccolo presynaptic cytomatrix protein	−1.34	0.0089
*CCDC30*	coiled-coil domain containing 30	−1.33	0.0115
*CDC40*	cell division cycle 40	−1.33	0.0303
*DNAJB5*	DnaJ (Hsp40) homolog, subfamily B, member 5	−1.33	0.0228
*RMI1*	RecQ mediated genome instability 1	−1.33	0.0495
*SLC25A36*	solute carrier family 25 (pyrimidine nucleotide carrier), member 36	−1.33	0.0231
*ZNF215*	zinc finger protein 215	−1.33	0.0353
*MAGEC3*	MAGE family member C3	−1.32	0.0298
*SLC35E4*	solute carrier family 35, member E4	−1.32	0.0151
*ATP2A2*	ATPase, Ca++ transporting, cardiac muscle, slow twitch 2	−1.31	0.0405
*FAM120A*	family with sequence similarity 120A	−1.31	0.0195
*FAM208B*	family with sequence similarity 208, member B	−1.31	0.0302
*KATNBL1*	katanin p80 subunit B-like 1	−1.31	0.049
*MFSD12*	major facilitator superfamily domain containing 12	−1.31	0.0399
*MT1A*	metallothionein 1A	−1.31	0.0471
*ZNF609*	zinc finger protein 609	−1.31	0.0383
*ZNF750*	zinc finger protein 750	−1.31	0.0259
*HLA-DOA*	major histocompatibility complex, class II, DO alpha	−1.3	0.0497
*KCNE4*	potassium channel, voltage gated subfamily E regulatory beta subunit 4	−1.3	0.0429
*LINC00656*	long intergenic non-protein coding RNA 656	−1.3	0.0252
*LINC01118*	long intergenic non-protein coding RNA 1118	−1.3	0.0228
*OR1M1*	olfactory receptor, family 1, subfamily M, member 1	−1.3	0.0127
*OSBPL8*	oxysterol binding protein-like 8	−1.3	0.0323
*RPAP2*	RNA polymerase II-associated protein 2	−1.3	0.0153
*ZNF257*	zinc finger protein 257	−1.3	0.0266

**Table 3:** Top enriched gene ontology clusters by the upregulated genes analysis by the DAVID.

Biological Process	
Term	Genes
GO:0045743~positive regulation of fibroblast growth factor receptor signaling pathway	*CTNNB1*, *NPTN*
GO:0014850~response to muscle activity	*METRNL*, *SRD5A1*
GO:0030539~male genitalia development	*CTNNB1*, *SRD5A1*
GO:0051493~regulation of cytoskeleton organization	*CAPN2*, *STMN1*
GO:0006367~transcription initiation from RNA polymerase II promoter	*TAF11*, *GTF2H2*, *NR1H2*
**Cellular Component**	
**Term**	**Genes**
GO:0005829~cytosol	*ACLY*, *DNAJC7*, *RAPGEF6*, *STRADA*, *USP6NL*, *WRD77*, *ANKFY1*, *CAPN2*, *CTNNB1*, *DCTN4*, *FN3KRP*, *GYG1*, *PSMB8*, *PPM1B*, *SIPA1*, *STMN1*
GO:0043209~myelin sheath	*WRD1*, *SRD5A1*, *UQCRC1*
GO:0005654~nucleoplasm	*ACLY*, *DNAJC7*, *RTCA*, *STRADA*, *TAF11*, *WRD77*, *CTNNB1*, *GTF2H2*, *ID1*, *NR1H2*, *PSMB8*
**Molecular Function**	
**Term**	**Genes**
GO:0047485~protein *N*-terminus binding	*TAF11*, *DCTN4*, *GTF2H2*, *ID1*

## Data Availability

All data generated or analyzed during this study are included in this published article and its supplementary information files. Microarray data are deposited in the Gene Expression Omnibus (GEO) under Accession Number: GSE148776 (https://www.ncbi.nlm.nih.gov/geo/query/acc.cgi?acc=GSE148776, accessed on 16 April 2020).
